# Drinking Water Salinity, Urinary Macro‐Mineral Excretions, and Blood Pressure in the Southwest Coastal Population of Bangladesh

**DOI:** 10.1161/JAHA.119.012007

**Published:** 2019-05-07

**Authors:** Abu Mohd Naser, Mahbubur Rahman, Leanne Unicomb, Solaiman Doza, Mohammed Shahid Gazi, Gazi Raisul Alam, Mohammed Rabiul Karim, Mohammad Nasir Uddin, Golam Kibria Khan, Kazi Matin Ahmed, Mohammad Shamsudduha, Shuchi Anand, K. M. Venkat Narayan, Howard H. Chang, Stephen P. Luby, Matthew O. Gribble, Thomas F. Clasen

**Affiliations:** ^1^ Emory Global Diabetes Research Center Hubert Department of Global Health Rollins School of Public Health Emory University Atlanta GA; ^2^ Department of Environmental Health Sciences Rollins School of Public Health Emory University Atlanta GA; ^3^ International Centre for Diarrhoeal Disease Research, Bangladesh (icddr,b) Dhaka Bangladesh; ^4^ Department of Geology University of Dhaka Bangladesh; ^5^ Institute for Risk and Disaster Reduction University College London London United Kingdom; ^6^ Division of Nephrology School of Medicine Stanford University Stanford CA; ^7^ Department of Biostatistics and Bioinformatics Rollins School of Public Health Emory University Atlanta GA; ^8^ Woods Institute for the Environment Stanford University Stanford CA; ^9^ Department of Epidemiology Rollins School of Public Health Emory University Atlanta GA

**Keywords:** blood pressure, calcium, drinking water salinity, magnesium, potassium, sodium, water salinity, Epidemiology, Diet and Nutrition, Hypertension

## Abstract

**Background:**

Sodium (Na^+^) in saline water may increase blood pressure (BP), but potassium (K^+^), calcium (Ca^2+^), and magnesium (Mg^2+^) may lower BP. We assessed the association between drinking water salinity and population BP.

**Methods and Results:**

We pooled 6487 BP measurements from 2 cohorts in coastal Bangladesh. We used multilevel linear models to estimate BP differences across water salinity categories: fresh water (electrical conductivity, <0.7 mS/cm), mild salinity (electrical conductivity ≥0.7 and <2 mS/cm), and moderate salinity (electrical conductivity ≥2 and <10 mS/cm). We assessed whether salinity categories were associated with hypertension using multilevel multinomial logistic models. Models included participant‐, household‐, and community‐level random intercepts. Models were adjusted for age, sex, body mass index (BMI), physical activity, smoking, household wealth, alcohol consumption, sleep hours, religion, and salt consumption. We evaluated the 24‐hour urinary minerals across salinity categories, and the associations between urinary minerals and BP using multilevel linear models. Compared with fresh water drinkers, mild‐salinity water drinkers had lower mean systolic BP (−1.55 [95% CI: −3.22–0.12] mm Hg) and lower mean diastolic BP (−1.26 [95% CI: −2.21–−0.32] mm Hg) adjusted models. The adjusted odds ratio among mild‐salinity water drinkers for stage 1 hypertension was 0.60 (95% CI: 0.43–0.84) and for stage 2 hypertension was 0.56 (95% CI: 0.46–0.89). Mild‐salinity water drinkers had high urinary Ca^2+^, and Mg^2+^, and both urinary Ca^2+^ and Mg^2+^ were associated with lower BP.

**Conclusions:**

Drinking mild‐salinity water was associated with lower BP, which can be explained by higher intake of Ca^2+^ and Mg^2+^ through saline water.

## Abstract 2

See Editorial Bispham and Nowak

**Clinical Perspective**

**What Is New?**

- Higher drinking water salinity or mineral contents are associated with higher urinary sodium, calcium, and magnesium concentrations.
- Blood pressure lowering effects of calcium and magnesium overweighed the blood pressure increasing effects of sodium, reflecting an overall inverse association between drinking water salinity, and blood pressure.

**What Are the Clinical Implications?**

- High sodium or low calcium or magnesium content in patients’ drinking water can increase their blood pressure and risks for hypertension.
- Adding calcium and magnesium to drinking water may be a useful strategy for reducing the population burden of hypertension when drinking water sources have low levels of these minerals.

## Introduction

Globally, >1 billion people living in coastal areas rely on groundwater as their principal water source.^1^ Nearly 204 million of them reside in areas that are affected by seawater intrusion,^2^ a process that increases groundwater salinity because of movement of the fresh‐saline groundwater interface towards the inland along the shores.^3^ Seawater intrusion will affect more coastal regions in the future because of increased volume of groundwater extraction to meet the population demand and global climate change such as change in precipitation patterns affecting groundwater recharge, decreased upstream river flow, frequent cyclones and sea‐level rise.^4^

Seawater intrusion causes mineralization of the groundwater.^5^ Communities in seawater intrusion affected areas drink brackish groundwater, rainwater, surface water (eg, pond water), or desalinated water.^6^ The salinity of these water sources varies as does the mineral concentrations; however, limited data exist on drinking water salinity, mineral intake, and cardiovascular health of the population. Drinking saline water has been associated with high sodium (Na^+^) intake,^7^ high blood pressure (BP),^8^ and high incidence of preeclampsia in seawater intrusion affected southwest coastal Bangladesh.^9^

Water salinity often refers to sodium chloride concentration, but in hydrogeology water salinity is measured as electrical conductivity (EC)—the ability of water to conduct electrical current or electrons where all dissolved ions are the conductors.^10^ The major cations contributing to water EC are Na^+^, calcium (Ca^2+^), potassium (K^+^), and magnesium (Mg^2+^)^11^—these are also the main macro‐minerals influencing human cardiovascular health. Most published studies from Bangladesh considered Na^+^ intake and urinary Na^+^ as a result of exposure to water salinity (Table 1),^7^, ^8^, ^9^, ^12^, ^13^ and therefore could not assess the health effects of other minerals present in brackish or saline water. Epidemiological studies, however, suggest that K^+^,^14^ Mg^2+^,^15^ and Ca^2+^,^16^ intake have inverse associations with BP and cardiovascular diseases. Drinking high‐salinity water may increase BP because of high Na^+^ concentration but may also lower BP if saline water contains high concentrations of K^+^, Mg^2+^, and Ca^2+^. In contrast, low‐salinity drinking water can reduce the intake of harmful Na^+^, but can also reduce intake of salubrious K^+^, Mg^2+^, and Ca^2+^. Data are limited on how all minerals together in saline water contribute to BP. We analyzed data from 2 studies to determine the association between drinking water salinity with BP, urinary Na^+^, K^+^, Ca^2+^, and Mg^2+^ excretion.

**Table 1:** Summary of Published Articles Examining Salinity and Blood Pressure in Southwest Coastal Bangladesh

Studies From Southwest Coastal Bangladesh	Salinity Measurement	Outcomes	Study Design	Study Duration	Geographical Coverage
Al Nahian et al^12^	Electrical conductivity	Hypertension	Longitudinal	Feb 2014 to Feb 2015 3 visits, 4 months apart	9 districts of coastal Bangladesh
Scheelbeek et al^13^	Na^+^ in water	Blood pressure of adult population	Cohort study	March 2013, March 2014, May 2014	3 subdistricts of same district
Talukder et al^8^	Electrical conductivity	Blood pressure	Cross‐sectional	May to June 2014	1 subdistrict
Khan et al^9^	Na^+^ in water	Preeclampsia	Case‐control	October 2009 to April 2011	1 subdistrict
Khan et al^7^	Electrical conductivity	Hypertension in pregnancy	Observational	October 2009 to March 2010	1 subdistrict

## Methods

### Study Population

**Figure 1:**
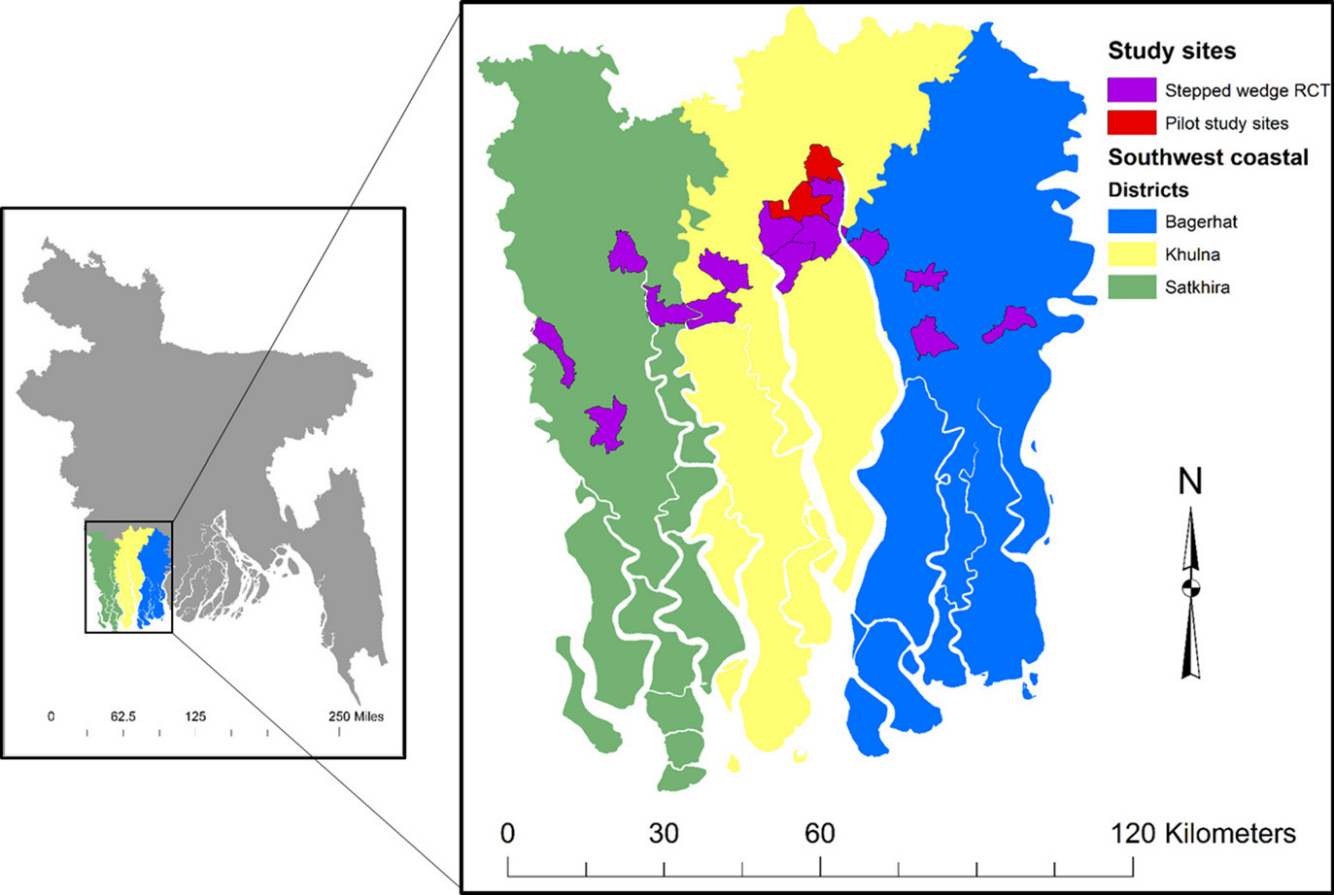
Map of the study sites. RCT indicates randomized controlled trial.

**Figure 2:**
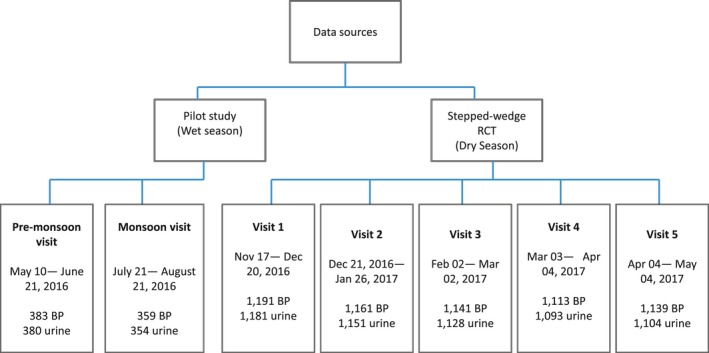
Data sources and study profiles. BP indicates blood pressure; RCT, randomized controlled trial.

The data that support the findings of this study are available from the corresponding author upon reasonable request. We pooled data from 2 studies led by the International Centre for Diarrhoeal Disease Research, Bangladesh across 3 seawater intrusion affected districts in southwest coastal Bangladesh (Figure 1). We pooled 6487 BP measurements and mineral concentrations of 6391 urine samples (Figure 2). The studies were implemented as part of a health impact evaluation of a drinking water salinity lowering intervention called managed aquifer recharge, a technology of artificially recharging brackish aquifers with rainwater and pond water to lower salinity.^17^ The first was an observational study that followed 383 participants from 166 households from 4 communities and visited each twice when participants drinking water salinity was low: once during the pre‐monsoon (May 10, 2016–June 20, 2016) and subsequently during the monsoon (July 20, 2016–August 20, 2016). The second study was a stepped‐wedge randomized trial (n=1191 from 542 households, followed for 5 visits) to assess the health impacts of water access across 16 communities during the dry season from December 2016 to April 2017 when participants drinking water salinity was high.^17^ The interval of both visits in the first study was 2 months and the interval between each successive visit of the second study was 1 month (Figure 2).

### Electrical Conductivity Measurement

During each visit, we recorded household‐reported primary drinking water sources used in the previous 24 hours and asked whether they had stored drinking water in their households. We collected available household stored drinking water samples and measured the temperature‐adjusted EC at 25°C during the visit using a Hanna Salinity meter (model: H198192, accuracy: ±1%). We calibrated the Salinity meters every 10 days.

### Blood Pressure Risk Factors

We collected data on demographics (age, sex, body mass index [BMI]), household assets, participant‐reported smoking status (never, current, and former smoker), and work‐related physical activity (vigorous, moderate, and sedentary). We also collected data on use of table salt during cooking (yes or no), consumption of additional table salt with food (yes or no), alcohol consumption (yes or no), hours of sleep (<6, ≥6 to <9, and ≥9 hours), and self‐reported disease status (hypertension, diabetes mellitus, and chronic kidney diseases) using a structured questionnaire. We used the World Health Organization (WHO) Global Physical Activity Questionnaire for determining participants physical activity status.^18^ Participants’ weight was measured in all visits using a Seca weight machine (model: 874‐1321009; accuracy: 0.05–0.1 kg, Hamburg, Germany) and height in 1 visit using a Shorr board (accuracy: 1/8” or 0.1 cm; Olney, Maryland).

### Outcomes

#### Blood pressure

During the same visit, participants’ systolic blood pressure (SBP) and diastolic blood pressure (DBP) were measured at each visit day (between 7.30 am and 2.00 pm) using an Omron HEM–907 (accuracy: within ±4 mm Hg, Kyoto, Japan) digital monitor usually by research staff of the same sex.^19^ Blood pressure was measured following WHO guidelines for BP measurement^20^ and the recommendations described by Pickering et al.^21^ Caffeine (tea, coffee, carbonated beverages), eating, heavy physical activities, and smoking were prohibited for 30 minutes before measuring BP. The blood pressure measurement procedure was described to participants who rested for at least 5 minutes on a chair in the sitting position with arms supported. An appropriately sized cuff based on mid‐upper arm circumference was used (small size cuff if mid‐upper arm circumference <22 cm; medium size cuff if mid‐upper arm circumference ≥22 to <32 cm; and large size if cuff ≥32 cm). BP was measured 3 times while in the sitting position; first left arm, then right arm, then again left arm—the arithmetic mean was used for analyses.

#### 24‐hour urine collection

We measured 24‐hour urine volume of the participants in all visits to measure the daily urinary excretions of minerals relevant to cardiovascular health, creatinine, and total protein. Twenty‐four‐hour urine volume also provided the information on daily water consumption by the participants. All participants received a 4‐L plastic container for 24‐hour urine collection and a mug to transfer the voided urine to the 4‐L plastic container. We instructed the participants to discard the first morning urine and start collecting from the second void,^22^ and to transfer all other voids of the day, and the next first morning.^23^ Volume of 24‐hour urine samples was measured at the household, and 15‐mL samples from the 4‐L plastic container were taken after stirring. We transported urine samples to a field laboratory at 2 to 8°C for processing, aliquoting, and analysis on the same day.

#### Twenty‐four‐hour urinary Na^+^, K^+^, Ca^2+^, Mg^2+^, creatinine, and total protein

Direct ion selective electrode methods, commonly used in clinical biochemistry laboratories with high agreement with the conventional flame photometer,^24^ were used to measure the urinary Na^+^ and K^+^ in all samples with a semi‐auto electrolyte analyzer (Biolyte 2000, Bio‐care Corporation, Taiwan, coefficient of variation: ±5%). Urinary Ca^2+^ and Mg^2+^ were measured by photometric titration methods using a semi‐auto biochemistry analyzer (Evolution 3000, BSI, Italy, coefficient of variation: <1%). Laboratory staff followed the manufacturer's guidelines for conditioning and calibration. We measured urine creatinine by a colorimetric method (Jaffe reaction). Urine total protein was measured using a colorimetric method by a semi‐auto biochemistry analyzer (Evolution 3000, BSI, Italy, coefficient of variation: <1%).

### Statistical Analyses

#### Descriptive statistics

We calculated mean and SD of approximately normally distributed variables, median and interquartile range of skewed variables, and proportions for categorical variables. We used the 2‐sample test of proportions or the Wilcoxon rank‐sum test, as applicable, to compare the proportions or medians with respect to reference group. We derived the household wealth score by principal component analysis using data for ownership of a refrigerator, television, mobile phones, motorcycle, bicycle, sewing machine, chair, table, wristwatch, wardrobe, wooden cot, motor pump, rice husking machine, motorized rickshaw, car, and access to electricity. We then categorized the wealth score into household wealth quintiles. We calculated pairwise Spearman correlations between drinking water EC and SBP.

#### Water salinity and blood pressure associations

The associations of concurrent water EC categories with mean SBP and DBP were modeled using multilevel linear models. EC categories were defined by the Food and Agricultural Organization of the United Nations: fresh water (EC <0.7 mS/cm), mild salinity (EC ≥0.7 and <2 mS/cm), and moderate salinity (EC ≥2 and <10 mS/cm).^25^ All regression models included 3‐level random intercepts to account for multilevel clustering of longitudinal visits within participants, participants within households, and households within communities. We estimated models using the maximum likelihood and reported cluster robust standard errors. We reported findings of unadjusted models (model 1); models adjusted for age, sex, BMI (model 2); and models that additionally adjusted for smoking, physical activities, alcohol consumption, consumption of additional table salt with food, sleep hours categories, religion, and household wealth (model 3). Age and BMI were used as continuous variables in the models, but other covariates were used as categorical variables (Table 2). Addition of table salt during cooking was not used in the model since 100% households reported to add salt during cooking; however, we adjusted models for the consumption of additional table salt with food.

**Table 2:** Characteristics of the Participants and Households at Enrollment

Characteristics	Drinking Water Electrical Conductivity (EC) Categories					
	Fresh Water (EC <0.7 mS/cm, n=547)	*P* Value	Mild‐Salinity Water (EC: 0.7 to <2 mS/cm, n=523)	*P* Value	Moderate‐Salinity Water (EC: 2–10 mS/cm, n=503)	*P* Value
Age (y), median (IQR)	40 (31–54)	Ref	41 (30–54)	0.900	40 (30–54)	0.672
Age categories, % (n)						
20 to <30 y	21 (117)	Ref	23 (122)	0.709	22 (110)	0.855
30 to <40 y	27 (150)	Ref	25 (130)	0.704	27 (137)	1.000
40 to <50 y	20 (112)	Ref	20 (105)	1.000	21 (105)	0.855
50 to <60 y	15 (82)	Ref	16 (82)	0.860	17 (87)	0.723
60 to <70 y	11 (58)	Ref	10 (54)	0.863	9 (43)	0.742
≥70 y	5 (28)	Ref	6 (30)	0.868	4 (21)	0.868
Male sex, % (n)	41 (226)	Ref	41 (214)	1.000	40 (203)	0.833
BMI, median (IQR)	22.3 (19.5–25)	Ref	21.6 (19.4–23.9)	0.006	21.4 (18.9–23.9)	<0.001
WHO BMI categories, % (n)						
Underweight (<18.5)	15 (79)	Ref	16 (81)	0.861	19 (94)	0.487
Normal weight (18.5 to <25)	59 (317)	Ref	67 (339)	0.034	64 (321)	0.194
Overweight (≥25 to <30)	22 (118)	Ref	15 (75)	0.229	14 (71)	0.175
Obese (≥30)	4 (23)	Ref	3 (14)	0.875	3 (13)	0.877
Smoking categories, % (n)						
Never	54 (294)	Ref	49 (258)	0.241	53 (267)	0.813
Former	9 (47)	Ref	12 (61)	0.617	8 (40)	0.868
Current	38 (206)	Ref	39 (204)	0.835	39 (196)	0.837
WHO work‐related physical activity, % (n)						
Sedentary	37 (205)	Ref	42 (219)	0.293	12 (59)	<0.001
Moderate^a^	39 (215)	Ref	34 (178)	0.306	71 (355)	<0.001
Vigorous^b^	23 (127)	Ref	24 (126)	<0.834	18 (89)	0.334
Urinary creatinine (mg/day), median (IQR)						
Male	1547 (1164–1951)	Ref	1471 (1123–1775)	0.051	1409 (1092–1787)	0.004
Female	1209 (948–1522)	Ref	1107 (881–1390)	0.012	1103 (928–1307)	<0.001
Household wealth categories, % (n)						
Lowest	14 (35)	Ref	18 (44)	0.016	29 (64)	0.093
Second	14 (35)	Ref	23 (55)	0.294	23 (51)	0.299
Third	18 (45)	Ref	23 (55)	0.540	19 (41)	0.905
Fourth	23 (56)	Ref	21 (51)	0.803	16 (34)	0.424
Highest	31 (75)	Ref	15 (36)	0.071	14 (30)	0.073
Added table salt with food	59 (322)	Ref	71 (370)		66 (333)	
Added table salt during cooking^c^ % (n)	100 (473)	Ref	100 (497)	1.000	100 (220)	1.000
Hours of sleep, % (n)						
<6 h	18 (96)	Ref	24 (126)	0.143	17 (86)	0.856
≥6 to <9 h	72 (395)	Ref	61 (318)	0.002	71 (357)	0.762
≥9 h	10 (56)	Ref	15 (79)	0.394	12 (60)	0.731
Alcohol consumption, % (n)	4 (22)	Ref	3 (15)	0.873	4 (19)	1.000
Religion, % (n)						
Hindu	53 (289)	Ref	55 (287)	0.630	46 (233)	0.112
Muslim	47 (258)	Ref	45 (236)	0.656	54 (270)	0.108
Self‐reported disease, % (n)						
Hypertension	18 (100)	Ref	12 (61)	0.310	15 (74)	0.600
Diabetes mellitus	5 (29)	Ref	4 (22)	0.866	5 (23)	1.000
Chronic kidney disease	2 (13)	Ref	2 (11)	1.000	2 (12)	1.000
Volume of 24‐h urine, median (IQR)^d^	2224 (1655–2861)	Ref	2030 (1515–2742)	0.045	2026 (1323–2530)	<0.001

BMI indicates body mass index; EC, electrical conductivity; IQR, interquartile range; WHO, World Health Organization.

a Work involves moderate‐intensity activity that causes small increases in breathing or heart rate such as brisk walking (or carrying light loads) for at least 10 minutes continuously.

b Work involves vigorous‐intensity activity that causes large increases in breathing or heart rate (carrying or lifting heavy loads, digging or construction work) for at least 10 minutes continuously.

c Data on use of salt during cooking were measured during the randomized‐controlled trial only. However. All households reported use of table salt during cooking, so this variable was not used for model adjustment.

d We noticed participants 24‐hour volume changed across different visits or seasons. Median 24‐hour urine volume was highest (2224 mL) during December (visit 1 of the stepped‐wedge trial), and the lowest (1764) during April (visit 5 of the stepped‐wedge trial). Median 24‐hour urine volume was 2222 mL, 2176 mL, and 1994 during January (visit 2), February (visit 3), and March (visit 4) in stepped‐wedge trail.

We initially included all person‐visits in models, and then conducted separate restricted analyses among participants who were non‐hypertensive and non‐diabetic based on their self‐reported information. In sensitivity analyses, we included participants who reported no history of chronic kidney disease and person‐visits when urinary total protein was <300 mg/day.

To evaluate how water salinity may influence the risk of hypertension categories among the study population, we used multilevel multinomial logistic models with 3‐level random intercepts described above. We used the 2017 American Heart Association guidelines for hypertension categories—normal BP (SBP <120 mm Hg and DBP <80 mm Hg); elevated BP (SBP 120–129 and DBP <80); stage 1 (SBP 130–139 or DBP 80–89), and stage 2 (SBP ≥140 or DBP ≥90) hypertension.^26^ We also conducted propensity score‐matched analyses of person‐visits from the high and low water EC distribution. We calculated that we needed a sample size of 1344 in each group to detect a difference of 2 mm Hg SBP between person‐visits from low and high water EC distribution groups (standard deviation of SBP=18.5, power 80%, type 1 error 5%, 2‐sided). We initially selected 1344 person‐visits for those with stored water from the lowest EC distribution, and twice as many (1344×2=2688) person‐visits for those with stored water from the highest EC distribution. Then we matched the 1344 lowest EC person‐visits on listed covariates using nearest‐neighbor matching by Mahalanobis distance to select matched 1344 person‐visits (out of 2644 person‐visits) from the highest EC distribution. Finally, 1344 person‐visits from the lowest EC distribution and matched 1344 person‐visits from the highest EC distribution were used in propensity score‐matched analyses. In the propensity‐score matched subpopulation, we used similar multilevel linear models described above, but modeled salinity as a binary variable (high versus low EC).

To illustrate whether the shape of the associations between water salinity and BP is non‐linear or not, we used restricted cubic splines plots of water EC adjusted for covariates.

#### Exploring the mechanisms of water salinity and blood pressure associations

To explore the mechanisms by which water EC influences BP, we initially examined whether water EC was associated with daily urinary excretions of macro‐minerals such as Na^+^, K^+^, Ca^2+^, and Mg^2+^ using similar multilevel linear models and 3‐level random intercepts. We then assessed how SBP or DBP changes because of 1 SD unit increase in 24‐hour urinary Na^+^ (1 SD=74 mmol/day), K^+^ (1 SD=15 mmol/day), Ca^2+^ (1 SD=3 mmol/day), and Mg^2+^ (1 SD=3 mmol/day) excretions using separate multilevel linear models. We used 3 approaches of modeling for detecting the associations between each of the urine minerals and BP—(1) all person‐visits; (2) all person‐visits but adjusted for urinary creatinine; and (3) restricted analyses among person‐visits with complete 24‐hour urine collection based on creatinine index ≥0.7.^27^ Creatinine index was defined as the ratio of measured versus predicted daily urinary creatinine.^27^ Predicted daily urinary creatinine was calculated using the Kawasaki formula.^28^

Several variables were missing in the data set (EC [n=56, 0.9%]; BMI [n=85, 1.3%]; wealth index [n=34, 0.5%]; Na^+^ [n=97, 1.5%], K^+^ [n=97, 1.5%], urine creatinine [n=97, 1.5%], Ca^2+^ [n=405, 6%], Mg^2+^ [n=831, 13%]). We assumed data are missing not at random and applied multiple imputation (n=40 imputations) using chained equations conditional on the listed variables in the fully adjusted models. In sensitivity analyses, we also reported the associations of concurrent water EC categories with mean SBP and DBP using multilevel linear models in complete cases without imputing missing data. All results were considered statistically significant at the 5% level. We performed statistical analyses in Stata, version 15.0 and R, version 3.3.1.

### Ethics

Informed written consent was obtained from all participants and household heads, and study protocols were approved by the Ethical Review Committee of International Centre for Diarrhoeal Disease Research, Bangladesh (PR‐15096).

## Results

### Study Participants and Characteristics

The median age and BMI of participants at enrollment were 40 (interquartile range: 31–54) years and 22 (interquartile range: 19–24) kg/m^2^ (Table 2). Most participants had normal weight (63%) as per WHO classification of BMI, were women (59%) and never smoked (52%).

### Water Salinity and Blood Pressure

**Figure 3:**
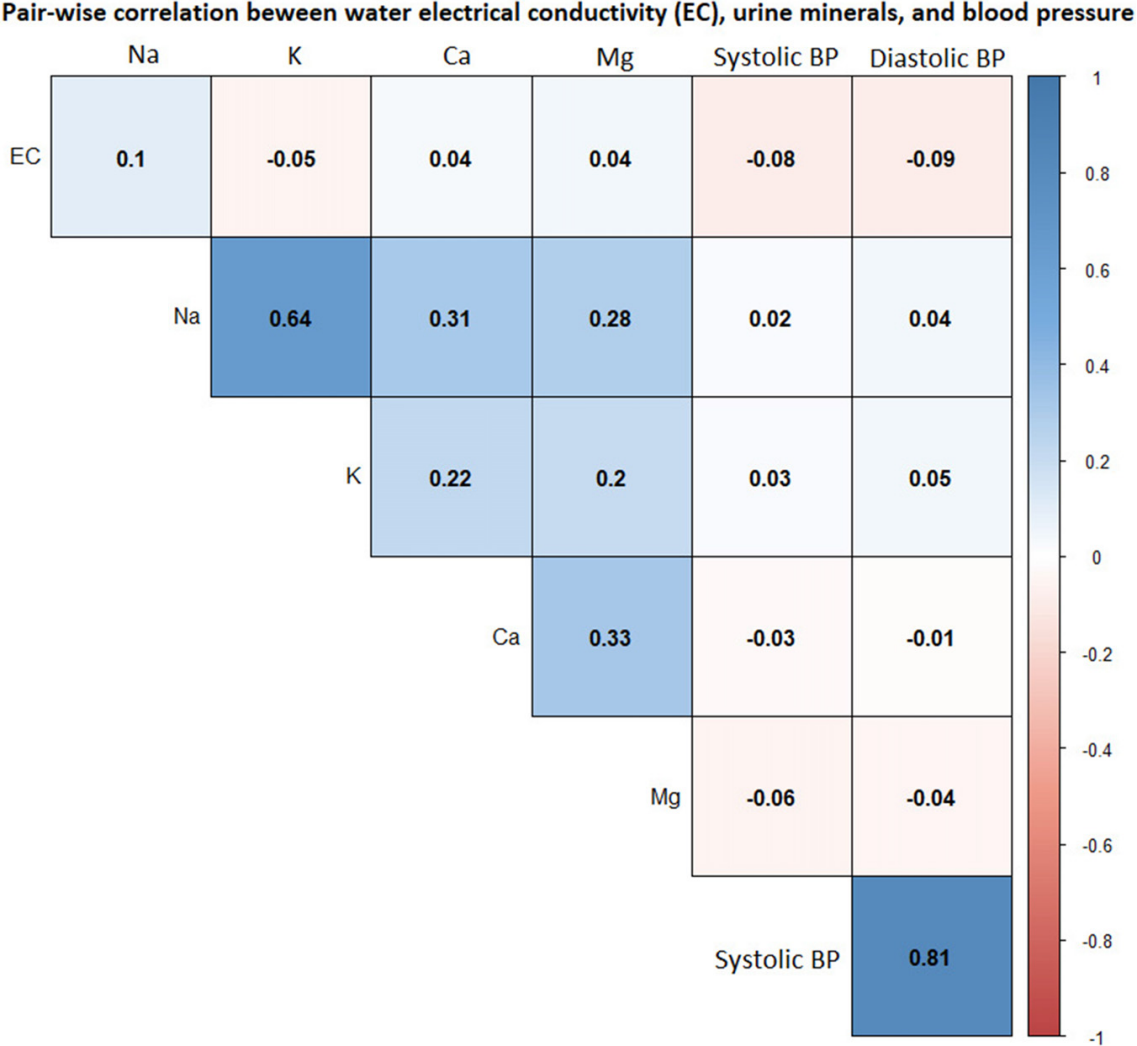
Pairwise correlation between households’ drinking water electrical conductivity, urinary minerals and systolic blood pressure in the pooled data. BP indicates blood pressure.

In all 6487 participant‐visits, 27% drank fresh water, 49% mild salinity, and 24% moderate‐salinity water. None of the water samples had high salinity (EC >10 mS/cm) based on the Food and Agricultural Organization classification. Spearman correlation coefficients suggests that participants whose drinking water EC was higher had lower SBP and DBP (Figure 3).

Compared with fresh water drinkers, mild‐salinity water drinkers had −1.55 (95% CI: −3.22–0.12) mm Hg SBP difference and −1.26 (95% CI: −2.21–−0.32) mm Hg DBP difference in the fully adjusted models (Table 3). Compared with fresh water drinkers, moderate‐saline water drinkers had −1.58 (95% CI: −3.13–−0.03) mm Hg SBP difference and −1.28 (95% CI: −2.10–−0.45) mm Hg DBP difference in the fully adjusted models (Table 3).

**Table 3:** Association Between Household Drinking Water Salinity Categories and Household Members’ BP

	Outcomes	Drinking Water Electrical Conductivity Categories		
		Fresh Water (EC: 0 to <0.7 mS/cm) (β, 95% CI)	Mild‐Salinity Water (EC: 0.7 to <2 mS/cm) (β, 95% CI)	Moderate‐Salinity Water (EC: 2.0–10 mS/cm) (β, 95% CI)
Person‐visits of all participants	Systolic BP			
	Model 1^a^	Reference	−1.63 (−3.25–0.00)	−1.73 (−3.25–−0.20)
	Model 2^b^	Reference	−1.59 (−3.25–0.07)	−1.64 (−3.17–−0.12)
	Model 3^c^	Reference	−1.55 (−3.22–0.12)	−1.58 (−3.13–−0.03)
	Diastolic BP			
	Model 1^a^	Reference	−1.33 (−2.22–−0.43)	−1.35 (−2.16–−0.55)
	Model 2^b^	Reference	−1.31 (−2.23–−0.38)	−1.32 (−2.13–−0.50)
	Model 3^c^	Reference	−1.26 (−2.21–−0.32)	−1.28 (−2.10–−0.45)
Person‐visits of non‐hypertensive and non‐diabetic participants	Systolic BP			
	Model 1^a^	Reference	−1.43 (−2.78–−0.07)	−1.70 (−3.13–−0.26)
	Model 2^b^	Reference	−1.38 (−2.77–0.02)	−1.61 (−3.07–−0.16)
	Model 3^c^	Reference	−1.34 (−2.75–0.06)	−1.56 (−3.03–−0.08)
	Diastolic BP			
	Model 1^a^	Reference	−1.12 (−1.98–−0.27)	−1.30 (−2.13–−0.48)
	Model 2^b^	Reference	−1.07 (−1.99–−0.17)	−1.25 (−2.10–−0.40)
	Model 3^c^	Reference	−1.04 (−1.97–−0.11)	−1.22 (−2.08–−0.36)

β refers to mean difference from the reference group. BP indicates blood pressure; EC, electrical conductivity.

a Unadjusted model.

b Adjusted for age, sex, and body mass index categories.

c Additionally adjusted for physical activities and smoking status, household wealth, alcohol consumption, sleep hours, religion, and consumption of additional table salt with food.

In restricted analyses among non‐hypertensive and non‐diabetic participants, we found that compared with fresh water drinkers, mild‐salinity water drinkers had −1.34 (95% CI: −2.75–0.06) mm Hg mean SBP difference and −1.04 (95% CI: −1.97–−0.11) mm Hg DBP difference. In restricted analyses, compared with fresh water drinkers, moderate‐salinity water drinkers had −1.56 (95% CI: −3.03–−0.08) mm Hg mean SBP difference and −1.22 (95% CI: −2.08–−0.36) mm Hg DBP difference in the fully adjusted models (Table 3).

Compared with the fresh water drinkers, the fully adjusted odds ratio for the mild‐salinity water drinkers was 0.60 (95% CI: 0.43–0.84) for stage 1 hypertension and 0.56 (95% CI: 0.46–0.89) for stage 2 hypertension. Compared with the fresh water drinkers, the fully adjusted odds ratio for the moderate‐salinity water drinkers for stage 1 hypertension was 0.77 (95% CI: 0.51–1.17) and for stage 2 hypertension was 0.61 (95% CI: 0.35–1.09) (Table 4).

**Table 4:** Odds Ratios of Having Elevated BP or Stage 1 or Stage 2 Hypertension, Relative to the Normal BP (SBP <120 mm Hg and DBP <80 mm Hg) Among Different Drinking Water Salinity Groups

	Water Salinity Categories	Elevated (SBP 120–129 and DBP <80)	Stage 1 Hypertension (SBP 130–139 or DBP 80–89)	Stage 2 Hypertension (SBP ≥140 or DBP ≥90)
Model 2	Fresh water (EC: <0.7 mS/cm)	Referent	Referent	Referent
	Mild‐salinity water (EC: 0.7 to <2 mS/cm)	0.88 (0.69–1.14)	0.58 (0.42–0.81)	0.54 (0.34–0.86)
	Moderate‐salinity water (EC: 2.0–10 mS/cm)	0.91 (0.68–1.22)	0.70 (0.47–1.04)	0.59 (0.34–1.04)
Model 3	Fresh water (EC: <0.7 mS/cm)	Referent	Referent	Referent
	Mild‐salinity water (EC: 0.7 to <2 mS/cm)	0.92 (0.71–1.18)	0.60 (0.43–0.84)	0.56 (0.46–0.89)
	Moderate‐salinity water (EC: 2.0–10 mS/cm)	0.96 (0.71–1.30)	0.77 (0.51–1.17)	0.61 (0.35–1.09)

Model 1 is unadjusted (we did not report model 1 as it did not converge for the multilevel multinomial outcome); model 2: adjusted for age, sex, and body mass index; model 3: additionally, adjusted for physical activities and smoking status, household wealth, alcohol consumption, sleep hours, religion, and consumption of additional table salt with food. DBP indicates diastolic blood pressure; EC, electrical conductivity; SBP, systolic blood pressure.

**Figure 4:**
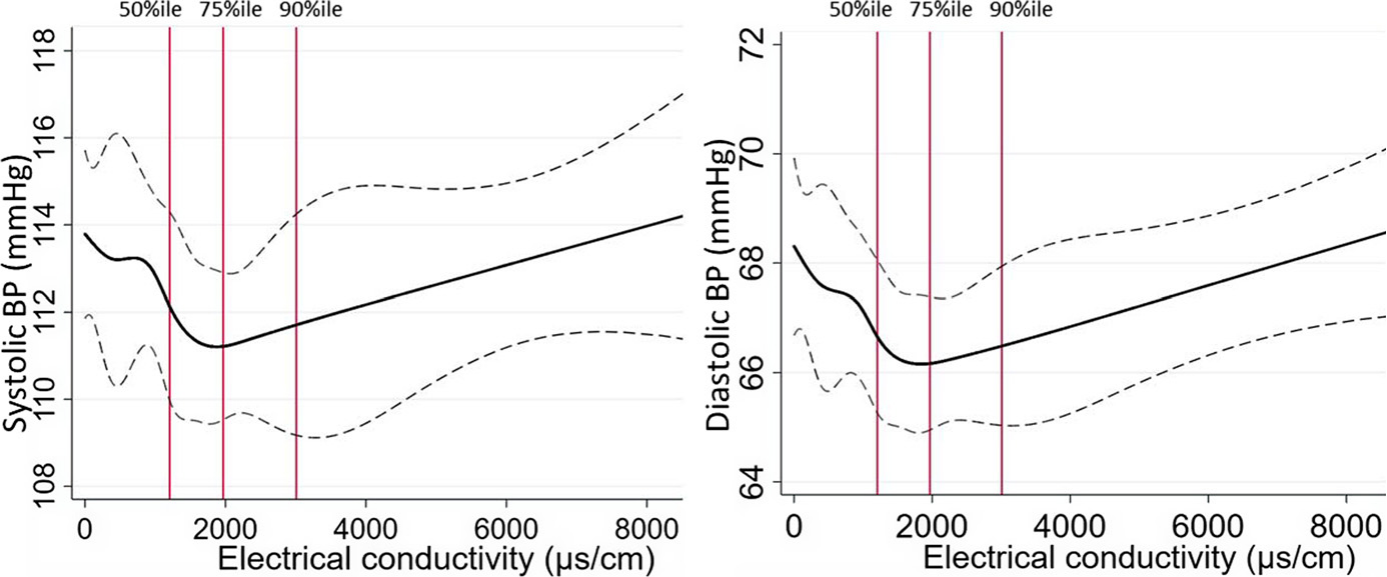
Restricted cubic spline plots (solid lines) and 95% CI (dashed lines) for the association between drinking water EC and blood pressure of the participants. Restricted cubic splines were plotted at EC cut points of 5th, 10th, 25th, 50th, 75th, 90th, and 95th percentile. Distribution of EC data at 50% (median), 75%, and 90% illustrated as red vertical lines. BP indicates blood pressure; EC, electrical conductivity.

In propensity score matching analyses, the matched high EC group had −1.64 (95% CI: −3.16–−0.12) mm Hg mean SBP difference and −1.54 (95% CI: −2.52–−0.58) mm Hg mean DBP difference in the fully adjusted models compared with the low EC group (Table 5). The water EC and BP restricted cubic spline plots suggest a non‐linear (Wald type test for non‐linearity, *P*<0.001 for SBP and <0.001 for DBP) and predominant negative association between drinking water EC and BP (Figure 4).

**Table 5:** Propensity Score Matched Analyses for the Association of Low Versus High Water EC Distribution on BP

Outcomes	Drinking Water Electrical Conductivity Categories	
	Low Salinity (EC: <197.9 μS/cm)	Matched High Salinity (EC: >1803 μS/cm)
Systolic BP (mean difference from the reference group)		
Model 1^a^	Reference	−1.87 (−3.30–−0.44)
Model 2^b^	Reference	−1.74 (−3.24–−0.24)
Model 3^c^	Reference	−1.64 (−3.16–−0.12)
Diastolic BP (mean difference from the reference group)		
Model 1^a^	Reference	−1.77 (−2.67–−0.87)
Model 2^b^	Reference	−1.65 (−2.60–−0.69)
Model 3^c^	Reference	−1.54 (−2.52–−0.58)

BP indicates blood pressure; EC, electrical conductivity.

a Unadjusted model.

b Adjusted for age, sex, and body mass index categories.

c Additionally adjusted for physical activities and smoking status, household wealth, alcohol consumption, sleep hours, religion, and consumption of additional table salt with food.

### Water Salinity and Urinary Cations

Both mild‐ and moderate‐salinity water drinkers had higher urinary Na^+^, Ca^2+^, and Mg^2+^ excretion than the fresh water drinkers (Table 6). Compared with fresh water drinkers, mild‐salinity water drinkers had 4.8 (95% CI: −1.0–10.7) mmol/day higher mean urinary Na^+^, 1.3 (95% CI: 1.2–1.5) mmol/day higher mean urinary Ca^2+^, and 1.2 (95% CI: 1.1–1.4) mmol/day higher mean urinary Mg^2+^ in the fully adjusted models (Table 7). Moderate‐salinity water drinkers had 16.7 (95% CI: 11.3–22.0) mmol/day higher mean urinary Na^+^, 1.2 (95% CI: 1.1–1.4) mmol/day higher mean urinary Ca^2+^, and 1.2 (95% CI: 1.1–1.4) mmol/day higher mean urinary Mg^2+^ in the fully adjusted model than the fresh water drinkers (Table 7).

**Table 6:** Urinary Na^+^, K^+^, Ca^2+^, and Mg^2+^ Excretion by Drinking Water Salinity Categories

Urinary Minerals	All Person‐Visits	Person‐Visits of Fresh Water Drinkers	Person‐Visits of Mild‐Salinity Water Drinkers	Person‐Visits of Moderate‐Salinity Water Drinkers
Urinary Na^+^				
Mean (SD)	165 (74)	155 (73)	166 (69)	172 (83)
Median (IQR)	154 (114–203)	144 (108–191)	158 (118–204)	160 (112–218)
Urinary K^+^				
Mean (SD)	34 (15)	34 (15)	35 (15)	33 (16)
Median (IQR)	32 (24–42)	32 (24–43)	33 (24–42)	30 (22–40)
Urinary Ca^2+^				
Mean (SD)	4 (3)	3.2 (2.8)	4.3 (3.1)	3.4 (3.0)
Median (IQR)	3 (1.6–5.1)	2.5 (1.3–4.3)	3.6 (2.1–5.7)	2.6 (1.3–4.6)
Urinary Mg^2+^				
Mean (SD)	4 (3)	3.3 (2.6)	4.0 (2.8)	4.0 (3.0)
Median (IQR)	3.3 (2.1–4.8)	2.8 (1.7–4.2)	3.6 (2.4–5.0)	3.5 (2.1–5.1)

IQR indicates interquartile range.

**Table 7:** Differences in Urinary Na^+^, K^+^, Ca^2+^ and Mg^2+^ Excretion Among Mild‐ and Moderate‐Salinity Water Drinkers Compared With Fresh Water Drinker When Adjusted for Different Level of Confounders

Urinary Cations	Drinking Water Electrical Conductivity (EC) Categories		
	Fresh Water (EC: 0 to <0.7 mS/cm)	Mild‐Salinity Water (EC: 0.7 to <2 mS/cm) (β, 95% CI)	Moderate‐SalinityWater (EC: 2.0–10 mS/cm) (β, 95% CI)
Urinary Na^+^			
Model 1^a^	Reference	4.6 (−1.4–10.5)	16.6 (11.3–21.9)
Model 2^b^	Reference	5.0 (−0.8–10.8)	16.9 (11.6–22.1)
Model 3^c^	Reference	4.8 (−1.0–10.7)	16.7 (11.5–22.0)
Urinary K^+^			
Model 1^a^	Reference	0.6 (−1.4–2.7)	0.0 (−2.00–2.00)
Model 2^b^	Reference	0.7 (−1.4–2.7)	0.1 (−1.9–2.0)
Model 3^c^	Reference	0.8 (−1.2–2.8)	0.2 (−1.8–2.1)
Urinary Ca^2+^			
Model 1^a^	Reference	1.4 (1.2–1.5)	1.2 (1.1–1.4)
Model 2^b^	Reference	1.4 (1.2–1.5)	1.2 (1.1–1.4)
Model 3^c^	Reference	1.3 (1.2–1.5)	1.2 (1.1–1.4)
Urinary Mg^2+^			
Model 1^a^	Reference	1.2 (1.1–1.4)	1.3 (1.1–1.4)
Model 2^b^	Reference	1.2 (1.1–1.4)	1.3 (1.1–1.4)
Model 3^c^	Reference	1.2 (1.1–1.4)	1.2 (1.1–1.4)

β refers to difference in mean urinary minerals between any water salinity and reference salinity group.

a Unadjusted model.

b Adjusted for age, sex, and body mass index categories.

c Additionally adjusted for physical activities and smoking status, household wealth, alcohol consumption, sleep hours, religion, and consumption of additional table salt with food.

### Urinary Cations and Blood Pressure

**Figure 5:**
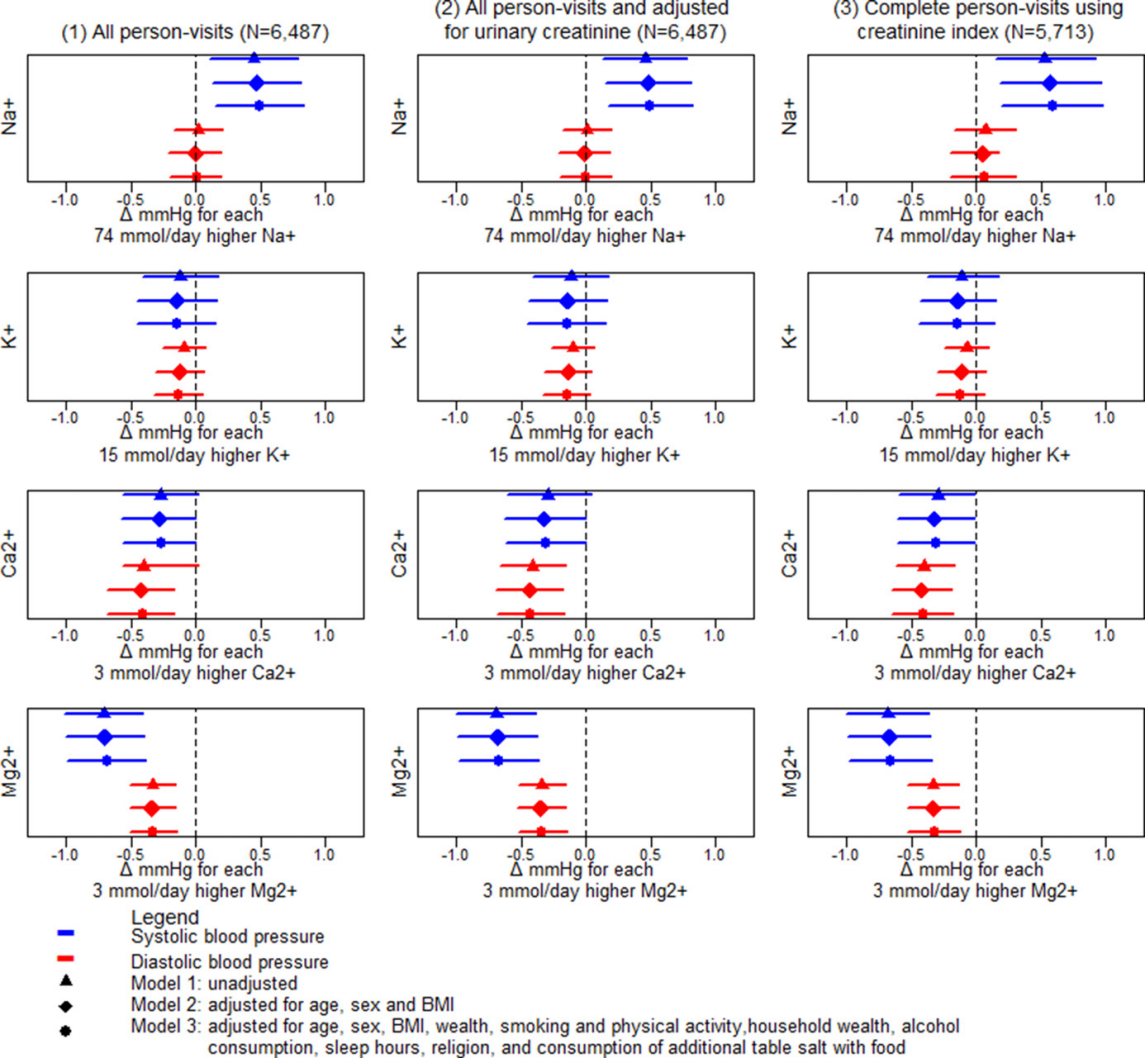
Association between 1 standard deviation higher urinary minerals and systolic and diastolic blood pressure considering (1) All person‐visits (2) All‐person‐visits and adjusting for urinary creatinine concentration, and (3) restricting the analyses among complete 24‐hour samples based on creatinine index. BMI indicates body mass index.

Higher urinary Na^+^ was associated with an increase in SBP, whereas higher urinary Ca^2+^ or urinary Mg^2+^ was associated with decreased SBP and DBP (Figure 5). A 74 mmol/day (1 SD) increase in urinary Na^+^ was associated with + 0.48 (95% CI: +0.14–+0.81) mm Hg higher mean SBP, and +0.00 (95% CI: −0.20–+0.20) mm Hg mean DBP difference in fully adjusted models. A 3 mmol/day (1 SD) increase in urinary Ca^2+^ was associated with −0.31 (95% CI: −0.01–−0.62) mm Hg lower mean SBP and −0.41 (95% CI: −0.16–−0.68) mm Hg lower mean DBP in fully adjusted models. A 3 mmol/day (1 SD) increase in urinary Mg^2+^ was associated with −0.7 (95% CI: −0.37–−0.97) mm Hg lower mean SBP and −0.3 (95% CI: −0.15–−0.51) mm Hg lower mean DBP in fully adjusted models (Figure 5). We found similar results in models additionally adjusted for urinary creatinine, or restricted among the complete 24‐hour urine collections (Figure 5).

### Sensitivity Analyses

Whenever we restricted the analyses to participants who did not report a history of chronic kidney disease and for person‐visits when urinary total protein was <300 mg/day, we found that mild‐salinity water drinkers had −1.32 (95% CI: −2.82–0.17) mm Hg SBP difference and −1.40 (95% CI: −2.25–−0.55) mm Hg DBP difference in the fully adjusted models compared with the fresh water drinkers (Table 8). Moderate saline water drinkers had −1.40 (95% CI: −3.14–0.34) mm Hg SBP difference and −1.29 (95% CI: −2.24–−0.33) mm Hg DBP difference in the fully adjusted models compared with the fresh water drinkers (Table 8).

**Table 8:** Sensitivity Analyses of Association Between Drinking Water Salinity Categories and Participants BP When Analyses was Restricted Among Participants With No Chronic Disease and Whose Urinary Protein was <300 mg/day

	Outcomes	Drinking Water Electrical Conductivity (EC) Categories		
		Fresh Water (EC: 0 to <0.7 mS/cm) (β, 95% CI)	Mild‐Salinity Water (EC: 0.7 to <2 mS/cm) (β, 95% CI)	Moderate‐Salinity Water (EC: 2.0–10 mS/cm) (β, 95% CI)
No chronic disease and urinary protein <300 mg/d	Systolic BP			
	Model 1^a^	Reference	−1.44 (−2.81–−0.08)	−1.59 (−3.23–0.05)
	Model 2^b^	Reference	−1.39 (−2.86–0.08)	−1.49 (−3.17–0.18)
	Model 3^c^	Reference	−1.32 (−2.82–0.17)	−1.40 (−3.14–0.34)
	Diastolic BP			
	Model 1^a^	Reference	−1.45 (−2.20–−0.70)	−1.37 (−2.29–−0.45)
	Model 2^b^	Reference	−1.46 (−2.28–−0.64)	−1.33 (−2.27–−0.40)
	Model 3^c^	Reference	−1.40 (−2.25–−0.55)	−1.29 (−2.24–−0.33)
Non‐hypertensive, non‐diabetic, no chronic kidney disease, and urinary protein <300 mg/d	Systolic BP			
	Model 1^a^	Reference	−1.35 (−2.51–−0.20)	−1.63 (−3.24–−0.02)
	Model 2^b^	Reference	−1.28 (−2.57–−0.00)	−1.54 (−3.24–0.16)
	Model 3^c^	Reference	−1.21 (−2.51–0.09)	−1.44 (−3.19–0.31)
	Diastolic BP			
	Model 1^a^	Reference	−1.33 (−2.07–−0.60)	−1.29 (−2.32–−0.25)
	Model 2^b^	Reference	−1.31 (−2.09–−0.55)	−1.26 (−2.31–−0.21)
	Model 3^c^	Reference	−1.26 (−2.07–−0.46)	−1.20 (−2.282–−0.12)

β refers to mean difference from the reference group. BP indicates blood pressure.

a Unadjusted model.

b Adjusted for age, sex, and body mass index categories.

c Additionally adjusted for physical activities and smoking status, household wealth, alcohol consumption, sleep hours, religion, and consumption of additional table salt with food.

In complete case analyses without missing data imputation, mild‐salinity water drinkers had −1.54 (95% CI: −3.32–0.23) mm Hg SBP difference and −1.30 (95% CI: −2.31–−0.30) mm Hg DBP difference in the fully adjusted models compared with the fresh water drinkers (Table 9). Moderate‐saline water drinkers had −1.36 (95% CI: −3.06–0.32) mm Hg SBP difference and −1.19 (95% CI: −2.07–−0.32) mm Hg DBP difference in the fully adjusted models compared with the fresh‐water drinkers (Table 9).

**Table 9:** Sensitivity Analyses of Association Between Drinking Water Salinity Categories and Participants BP Without Missing Data Imputation

	Outcomes	Drinking Water Electrical Conductivity Categories		
		Fresh Water (EC: 0 to <0.7 mS/cm) (β, 95% CI)	Mild‐Salinity Water (EC: 0.7 to <2 mS/cm) (β, 95% CI)	Moderate‐Salinity Water (EC: 2.0–10 mS/cm) (β, 95% CI)
Person‐visits of all participants	Systolic BP			
	Model 1^a^	Reference	−1.62 (−3.27–0.02)	−1.52 (−3.13–−0.08)
	Model 2^b^	Reference	−1.59 (−3.34–0.16)	−1.47 (−3.10–0.16)
	Model 3^c^	Reference	−1.54 (−3.32–0.23)	−1.36 (−3.06–0.32)
	Diastolic BP			
	Model 1^a^	Reference	−1.33 (−2.24–−0.42)	−1.24 (−2.24–−0.42)
	Model 2^b^	Reference	−1.35 (−2.33–−0.37)	−1.24 (−2.10–−0.39)
	Model 3^c^	Reference	−1.30 (−2.31–−0.30)	−1.19 (−2.07–−0.32)
Person‐visits of non‐hypertensive and non‐diabetic participants	Systolic BP			
	Model 1^a^	Reference	−1.45 (−2.81–−0.09)	−1.56 (−3.04–−0.08)
	Model 2^b^	Reference	−1.39 (−2.83–0.04)	−1.50 (−3.00–0.00)
	Model 3^c^	Reference	−1.34 (−2.79–0.11)	−1.41 (−2.96–0.13)
	Diastolic BP			
	Model 1^a^	Reference	−1.14 (−2.00–−0.28)	−1.21 (−2.04–−0.38)
	Model 2^b^	Reference	−1.14 (−2.04–−0.24)	−1.21 (−2.05–−0.36)
	Model 3^c^	Reference	−1.09 (−2.01–−0.17)	−1.15 (−2.02–−0.30)

β refers to mean difference from the reference group. BP indicates blood pressure.

a Unadjusted model.

b Adjusted for age, sex, and body mass index categories.

c Additionally adjusted for physical activities and smoking status, household wealth, alcohol consumption, sleep hours, religion, and consumption of additional table salt with food.

## Discussion

Our analyses suggest that in seawater intrusion affected southwest coastal Bangladesh, drinking mild‐salinity water was associated with lower BP. We also found drinking mild‐salinity water was associated with lower risks of stage 1 and stage 2 hypertension among the study population.

We suspect that the effects of drinking mild‐ and moderate‐salinity water on BP may be attributable to high Ca^2+^ and Mg^2+^ present in saline water. Similar to other study findings conducted in southwest coastal Bangladesh,^9^, ^12^, ^13^, ^29^ we found that drinking mild‐ and moderate‐salinity water was associated with higher urinary Na^+^, and higher urinary Na^+^ was associated with higher SBP. We additionally found that drinking mild‐ and moderate‐salinity water EC was associated with higher urinary Ca^2+^ and Mg^2+^, and both urinary minerals were associated with lower SBP and DBP. We hypothesize that the BP‐lowering effects of Ca^2+^ and Mg^2+^ counteracted the harmful effects of Na^+^, reflected by the overall inverse association between drinking mild‐ and moderate‐salinity water EC and BP. Similarly, BP‐lowering effects of drinking water rich in Ca^2+^ and Mg^2+^ have been observed across many regions of the world.^30^, ^31^ Drinking water rich in Ca^2+^ and Mg^2+^ was associated with reduced cardiovascular and cerebrovascular mortality.^32^, ^33^

These findings may be generalizable to other seawater intrusion‐affected coastal regions. The predominant cations in seawater are Na^+^, Ca^2+^, and Mg^2+^.^34^ These minerals have been reported in high concentrations in groundwater of seawater intrusion affected coastal regions across the world including deltas,^35^ arid or semi‐arid regions,^5^ peninsula,^36^ and islands.^37^ A hydro‐geological survey in Bangladesh suggests that groundwater hardness—a measure of Ca and Mg salts—is the highest in seawater intrusion‐affected southwest Bangladesh,^38^ where the groundwater is of the Na‐Ca‐Mg‐HCO_3_‐Cl type. When communities in seawater intrusion‐affected areas drink Na^+^, Ca^2+^, and Mg^2+^ rich water, their intakes of these minerals increase, evident in our study as high urinary Na^+^, Ca^2+^, and Mg^2+^ concentrations. People in Bangladesh have lower intake of Ca and Mg through their regular diet,^39^ therefore, drinking water can be an important source of these minerals. In settings where communities have higher dietary intake of Ca and Mg, intake of these minerals though drinking water may be less beneficial.

Experimental studies suggest that Ca^2+^ and Mg^2+^ can counterbalance the effect of Na^+^ on BP.^15^ Entry of Na^+^ across the cell membrane of vascular smooth muscle precedes smooth muscle contraction that increases vascular tone and BP.^40^ In contrast, Ca^2+^ and Mg^2+^ decrease BP by stabilizing the cell membrane of the vascular smooth muscle by binding to the plasma membrane,^41^, ^42^ which in turn interferes with the ionic conductance that diminishes vascular tone.^43^ Ca^2+^ and Mg^2+^ concentrations below physiological levels destabilizes the cell membrane, causing greater Na^+^ entry across the cell membrane and attenuates smooth muscle contraction.^44^ Increased dietary intake of Ca^2+^ and Mg^2+^ also facilitates urinary excretion of Na^+^ by a variety of mechanisms including increased release of atrial natriuretic peptide, reduced sympathetic outflow and interference with Na^+^ re‐absorption by kidneys.^45^, ^46^

Our analyses have several key limitations. First, we were unable to measure the concentrations of individual minerals in water because of high costs. This precludes the understanding of exact mineral exposure through high EC water. We also lack bioavailability data for minerals from drinking water, however, studies support high bioavailability of Ca and Mg from drinking water.^47^ We also did not collect mineral intake data of the participants through diet, which precludes our understanding of what percentage of urinary mineral concentrations were coming from food or drinking water. Although 24‐hour urine collection is the ideal method for urinary mineral measurements,^27^ it may be biased by over‐ or under‐collection of urine samples.^27^ We attempted to minimize bias by analyzing data from participants with complete 24‐hour urine collection based on the urinary creatinine index.^23^ Several studies have reported Na^+^ induces calciuria or Ca^2+^ excretion through urine.^48^ Therefore, high urinary Ca^2+^ among study participants could be partially because of the influence of Na^+^ on kidneys in addition to Ca^2+^ intake through high EC water. Whenever we restricted the analyses excluding the self‐reported chronic kidney participants and those with >300 mg/day urinary total protein, the findings were slightly attenuated. We only had a few self‐reported chronic kidney participants, but we were unable to measure renal function of the participants using serum creatinine or estimated glomerular filtration rate as we did not collect blood samples of the participants. We had few high‐salinity water drinkers thereby limiting insight on the shape of the EC and BP dose response curve, however, this may reflect community behavior as many people report that high EC water has a disagreeable taste. Moderate‐salinity water drinkers had higher urinary Na^+^ than the mild‐salinity water drinkers but no differences were observed for urinary Mg^2+^. High‐salinity water drinkers may have hypertension due to increased Na intake, but we could not assess this. BP has a diurnal variation and participants whose BP was measured in the morning may had higher BP than participants whose BP was measured around noon or afternoon.^49^ We did not collect the exact time of BP measurement and thereby were unable to control for it, which likely introduced measurement error for BP.

The nuanced effects of drinking water salinity on blood pressure in Bangladesh are consistent with other observations. Blood Mg concentration was lower and mortality after hospitalization was higher in areas served by desalinated water in Israel compared with areas served by non‐desalinated water.^50^ Populations exposed to desalinated water had higher risks for ischemic heart disease.^51^ Those that have low‐salinity drinking water (eg, rainwater, desalinated water, reverse osmosis water) should explore adding calcium and magnesium to their water sources to reduce the risks of blood pressure and cardiovascular diseases.^52^ Similarly, adding calcium and magnesium to drinking water may be a useful strategy for reducing the population burden of hypertension when drinking water sources have low levels of these minerals. Ensuring optimum concentrations of Ca^2+^ and Mg^2+^ in drinking water may be an important public health and nutritional intervention to ensure fulfillment of daily requirements of these essential macro‐minerals since evidence suggests that globally concentrations of these minerals are decreasing in the diet.^53^, ^54^

## Sources of Funding

This research was funded by Wellcome Trust, UK, Our Planet, Our Health Award (Grant # 106871/Z/15/Z).

## Disclosures

None.
